# Properties of Parallel Tetramolecular G-Quadruplex Carrying *N*-Acetylgalactosamine as Potential Enhancer for Oligonucleotide Delivery to Hepatocytes

**DOI:** 10.3390/molecules27123944

**Published:** 2022-06-20

**Authors:** Anna Clua, Santiago Grijalvo, Namrata Erande, Swati Gupta, Kristina Yucius, Raimundo Gargallo, Stefania Mazzini, Muthiah Manoharan, Ramon Eritja

**Affiliations:** 1 Institute for Advanced Chemistry of Catalonia (IQAC-CSIC), Jordi Girona 18-26, E-08034 Barcelona, Spain; acvtnt@cid.csic.es (A.C.); sgrgma@cid.csic.es (S.G.); 2 Networking Center on Bioengineering, Biomaterials and Nanomedicine (CIBER-BBN), E-08034 Barcelona, Spain; 3 Alnylam Pharmaceuticals, 300 Third Street, Cambridge, MA 02142, USA; nd.erande@gmail.com (N.E.); sgupta@alnylam.com (S.G.); kyucius@alnylam.com (K.Y.); mmanoharan@alnylam.com (M.M.); 4 Department of Chemical Engineering and Analytical Chemistry, University of Barcelona, Martí i Franquès 1-11, E-08028 Barcelona, Spain; raimon_gargallo@ub.edu; 5 DEFENS-Dipartimento Di Scienze per Gli Alimenti, la Nutrizione e l’Ambiente, Università degli Studi di Milano, Via Celoria, 2, 20133 Milan, Italy; stefania.mazzini@unimi.it

**Keywords:** G-quadruplex, *N*-acetylgalactosamine, antisense, oligonucleotide conjugates, asialoglycoprotein receptor, luciferase gene, gapmers

## Abstract

The development of oligonucleotide conjugates for in vivo targeting is one of the most exciting areas for oligonucleotide therapeutics. A major breakthrough in this field was the development of multifunctional GalNAc-oligonucleotides with high affinity to asialoglycoprotein receptors (ASGPR) that directed therapeutic oligonucleotides to hepatocytes. In the present study, we explore the use of G-rich sequences functionalized with one unit of GalNAc at the 3′-end for the formation of tetrameric GalNAc nanostructures upon formation of a parallel G-quadruplex. These compounds are expected to facilitate the synthetic protocols by providing the multifunctionality needed for the binding to ASGPR. To this end, several G-rich oligonucleotides carrying a TGGGGGGT sequence at the 3′-end functionalized with one molecule of *N*-acetylgalactosamine (GalNAc) were synthesized together with appropriate control sequences. The formation of a self-assembled parallel G-quadruplex was confirmed through various biophysical techniques such as circular dichroism, nuclear magnetic resonance, polyacrylamide electrophoresis and denaturation curves. Binding experiments to ASGPR show that the size and the relative position of the therapeutic cargo are critical for the binding of these nanostructures. The biological properties of the resulting parallel G-quadruplex were evaluated demonstrating the absence of the toxicity in cell lines. The internalization preferences of GalNAc-quadruplexes to hepatic cells were also demonstrated as well as the enhancement of the luciferase inhibition using the luciferase assay in HepG2 cell lines versus HeLa cells. All together, we demonstrate that tetramerization of G-rich oligonucleotide is a novel and simple route to obtain the beneficial effects of multivalent *N*-acetylgalactosamine functionalization.

## 1. Introduction

The development of oligonucleotides for therapeutic use has entered an expansive phase with an arsenal of more than a dozen drugs based on synthetic oligonucleotides [1,2]. This is especially evident in the last three years, with an increase of new oligonucleotide approvals per year being one of the most prominent biomolecules approved for human use [3]. Most of these therapeutic drugs are antisense oligonucleotides [4] or siRNA duplexes [5] that target overexpressed messenger RNA (mRNA). Others are aptamers that target overexpressed proteins, such as pegaptanib that target vascular endothelial growth factor (VEGF) [6]. However, soon it is expected that new oligonucleotides will be used for modulating other important RNA targets such as microRNA [7] and long non coding (lnc) RNA [8].

In order to achieve the desired therapeutic action, oligonucleotides should penetrate inside the cells and bind readily to their complementary sequence before exonuclease activity degrades them. A large research activity is being made to design modified oligonucleotides with enhanced nuclease resistance and cellular uptake as well as higher selectivity to target tissues [1,5]. However, the delivery of modified DNA and RNA is the major bottleneck in the development of nucleic acids as drugs [9]. The importance of this issue can be demonstrated in the newly approved siRNAs, Givosiran [10] and Inclisiran [11], which are functionalized with triantennary *N*-acetylgalactosamine (GalNAc) allowing the drug to maintain their silencing properties for 6 months [11]. In these conjugates, the 3′ terminus of the siRNA sense strand is linked to three molecules of GalNAc that have a high binding affinity to the asialoglycoprotein receptor [12], or by successive additions of a GalNAc monomer [13,14]. The asialoglycoprotein receptor is mainly found in hepatocytes exhibiting a high affinity for galactose glycoproteins and trivalent GalNAc oligonucleotides with a rapid internalization rate via the clathrin-mediated pathway [12,15].

Another potential interest of oligonucleotides is the possibility of using their singular non-canonical structures for protein binding, especially guanine-rich oligonucleotide that may form G-quadruplex, as some of them have antiviral, anticancer and anticoagulant properties [16,17,18]. The antiviral properties usually result from the binding of the quadruplex to viral proteins [19]. These aptamers have also been used for targeted drug delivery as they have affinity to proteins that are overexpressed in cancer cells, such as AS1411 that binds nucleolin [18,20]. Recently, antisense oligonucleotides were attached to simple G-rich sequences to form parallel G-quadruplex structures. These simple DNA scaffolds were efficient in delivering the antisense oligonucleotide cargo inside the cells without the presence of cationic lipids as transfecting reagents [21,22]. Moreover, parallel G-quadruplexes have been used for increased delivery of antiproliferative floxuridine oligonucleotides upon tetramerization of G-rich sequences [23], as some cancer cells have abundant G-quadruplex binding proteins.

**Scheme 1:**
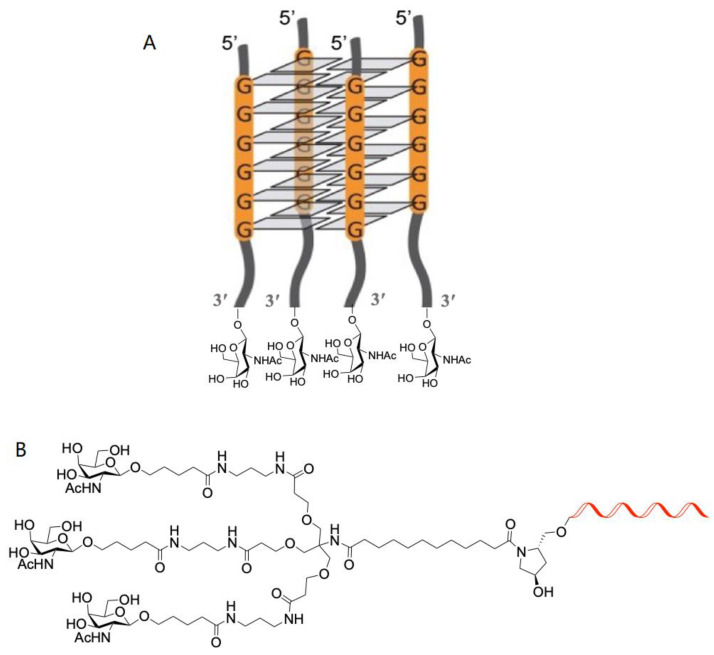
Chemical schemes of potential multifunctional GalNAc derivatives including (**A**) tetrameric G-quadruplexes studied in this work and (**B**) the triantennary GalNAc.

In the present study, we explore the use of G-rich sequences functionalized with one unit of GalNAc at the 3′-end for the formation of tetrameric GalNAc nanostructures upon formation of parallel G-quadruplex. This study was aimed to provide an alternative method to obtain multifunctional GalNAc-oligonucleotides with a more simplified synthetic method (Scheme 1, Figure S1). To this end, we prepare G-rich oligonucleotides carrying a TGGGGGGT sequence at the 3′-end functionalized with one molecule of *N*-acetylgalactosamine (GalNAc). These compounds are easier to synthesize than the “standard” triantennary GalNAc. We aim to study if the association of four strands produces G-quadruplex tetrameric GalNAc derivatives that may provide a similar affinity to ASGPR than the “standard” triantennary-GalNAc. The formation of self-assembled parallel G-quadruplexes is studied through various biophysical techniques such as circular dichroism, nuclear magnetic resonance, polyacrylamide electrophoresis and denaturation curves. Binding experiments to ASGPR show that the size and the relative position of the therapeutic cargo are important for the binding of these nanostructures to ASGPR. All together we demonstrate that tetramerization of G-rich sequences is a novel strategy to facilitate specific cellular uptake of short single-stranded oligonucleotides to hepatocytes including antisense gapmers directed against luciferase.

## 2. Results

### 2.1. Oligonucleotides

**Scheme 2:**
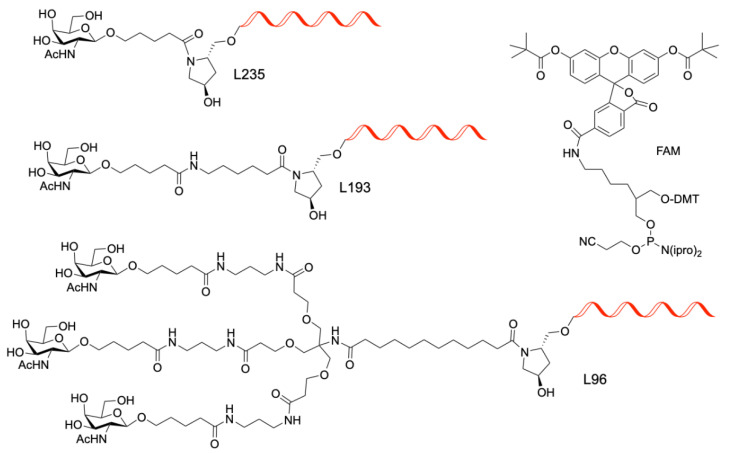
Chemical structures of the GalNAc and FAM ligands used in this work.

Table 1 and Table 2 show the oligonucleotides prepared in this study indicating the backbone and the presence or absence of *N*-acetylgalactosamine modification at the 3′-position. The first group of derivatives is derived from a 17 mer (TTGGGGGGTACAGTGCA) sequence (Table 1). This sequence is present in the HIV-1 genome [24,25]. Lipid-modified oligonucleotide derivatives or lipoquads have antiviral properties (both against HIV [26] and HCV [17]) as inhibitors of viral entry. These sequences contain natural phosphodiester backbone (H2–H4). Two different GalNAc derivatives with different length were studied (L193 (longer), and L235 (shorter), Scheme 2). The synthesis of the appropriate solid supports functionalized with these GalNAc derivatives has been reported [13]. Some of these oligonucleotides (H5, H6) were fluorescein labeled to visualize cellular uptake by flow cytometry. Control oligonucleotides include unmodified control sequence (H1) and A-rich analogues (H3, H5). Finally, some oligonucleotides contained two phosphorothioate linkages at the terminal 3′ and 5′-ends (H8–H11). The A-rich oligonucleotide H12 carrying the “classical” triantennary GalNAc ligand L96 was also made for comparison purposes during the evaluation of the binding of G-rich oligonucleotides to the asialoglycoprotein (ASGPR) receptor.

The second group of oligonucleotides combined a gapmer sequence against *Renilla* luciferase (5′-CsGsUsUsTsCsCsTsTsTsGsTsTsCsUsGsGsA-3′) [27] carrying 2′-O-methyl-RNA modifications at the last four positions of the 3′- and 5′-ends (underlined) and phosphorothioate linkages with a G-quadruplex forming TG_6_T sequence. These include two different spatial distributions. In one oligonucleotide, gapmer was located at the 5′-end and TG_6_T sequence was near the 3′-end (L3), and in another oligonucleotide, TG_6_T sequence was located at the 5′-end and the gapmer sequences were located near the 3′-end (L4). In addition, we prepared a fluorescein labeled oligonucleotide (L7), an oligonucleotide carrying a scrambled sequence instead of the luciferase sequence (L6), unmodified antisense (L1) and gapmer (L2) antisense control sequences and control T-rich oligonucleotides unable to form G-quadruplex (L5, L8). All these sequences except L8 contained phosphorothioate linkages in the antisense/gapmer moiety, but they contained the natural phosphodiester linkages in the TG_6_T or T_4_ sequence.

A third group of oligonucleotides (Table S2) were derived from the therapeutic siRNA against the mouse transthyretin (mTTR) gene [12]. In this case, they were a pair of RNA strands that were highly modified with 2′-O-methyl-RNA and 2′-fluoro-RNA nucleotides as well as two phosphorothioate linkages at the two last 5′-terminal positions. The TG_6_T sequence was added to the 3′-position of the sense strand ending with the L193 GalNAc ligand in oligonucleotide R2. Oligonucleotide R1 was a control sense sequence carrying the sense strand and the triantennary GalNAc (L96) ligand. The oligonucleotide R3 was the antisense strand complementary to R1 and R2 oligonucleotides to obtain the double-stranded form.

### 2.2. Characterization of G-Quadruplex Formation

First of all, the formation of a parallel G-quadruplex was studied by biophysical techniques, such as circular dichroism (CD), gel electrophoresis and nuclear magnetic resonance (NMR).

#### 2.2.1. Characterization of G-Quadruplex Formation by CD

**Figure 1:**
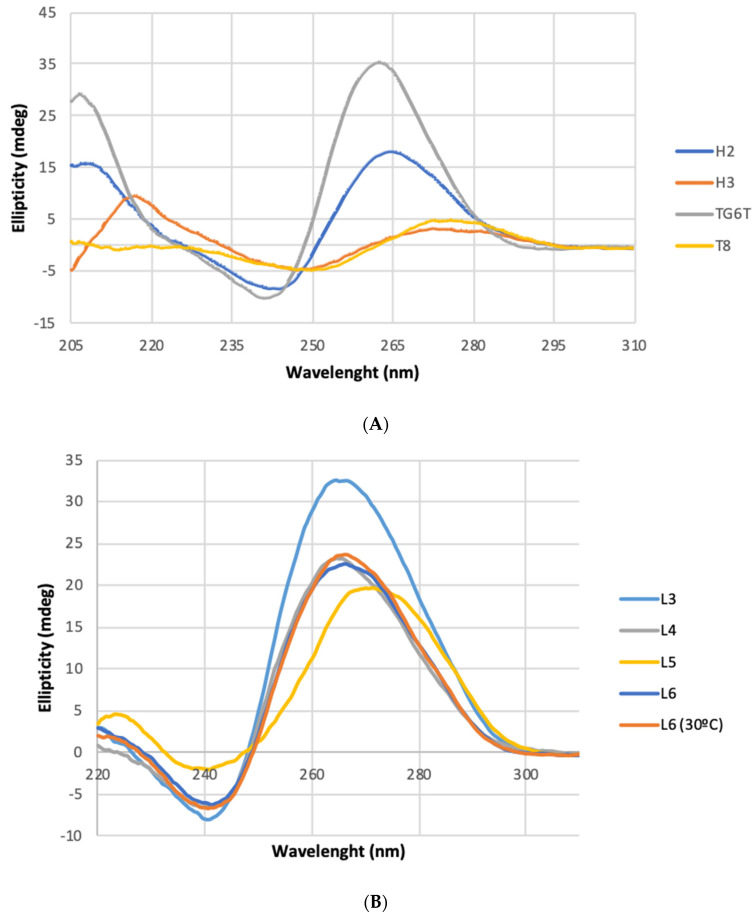
(**A**) CD spectra of G-rich sequence (G-PO-HCV-L235, H2) versus A-rich sequence (A-PO-HCV-L235, H3). The CD spectra of TG_6_T and T_8_ are included for comparison. Buffer conditions PBS 1×. (**B**) CD spectra of G-rich sequences carrying the antiluciferase sequence (L3, L4, L6) versus the control T-rich sequence (L5) Buffer conditions PBS 1×.

Figure 1A shows the CD spectra of the G–quadruplex sequence H2 (G-PO-HCV-L235) compared with the A-rich sequence H3 (A-PO-HCV-L235) from 205 to 310 nm. The CD spectra of control oligonucleotides TG_6_T (tetrameric G-quadruplex) and T_8_ (single stranded) were added for comparison. The G-rich oligonucleotides (H2 and TG_6_T) showed intense positive bands at around 210 and 261 nm characteristic of a parallel G-quadruplex [28], while the A-rich sequence (H3) and T_8_ presented a maximum at 274 nm. In addition the CD spectrum of the G-rich sequences H2, H4, H10 and H11 maintain the same shape unaltered up to 90 °C (Figure S2) indicating that the parallel G-quadruplex structure is thermally stable at all studied temperatures. This thermal stability is in agreement with the reported values for the well-studied tetrameric parallel G-quadruplex obtained with TG_n_T oligonucleotides [21,22,23,29]. TG_4_T melting can only be observed in sodium buffers, but not in potassium buffers [21,22,23,29]. The melting temperatures of the six tetrads quadruplex (TG_6_T derivatives) cannot be measured because they do not melt even at temperatures higher than 80 °C [29]. For example, TG_4_T melts at 59.4 °C, but TG_6_T cannot be measured (>80 °C) in 10 mM sodium cacodylate buffer with 0.15 M NaCl (pH 7.2) [21,22]. For the modified TG_6_T oligonucleotides carrying an antisense oligonucleotide with lipids, or floxuridine or positively charged ligands, we cannot observe any melting behavior as G-quadruplex were stable up to 80 °C [21,22,23].

CD spectra of the rest of the G-rich oligonucleotides show similar positive bands at 265 nm and negative bands near 240 nm (Figure 1B, Figures S2 and S3) characteristic of a parallel G-quadruplex. Oligonucleotide L5 carrying T’s instead of G’s showed a maximum at around 272 nm (Figure 1B and Figure S3).

#### 2.2.2. Characterization of G-Quadruplex Formation by NMR

**Figure 2:**
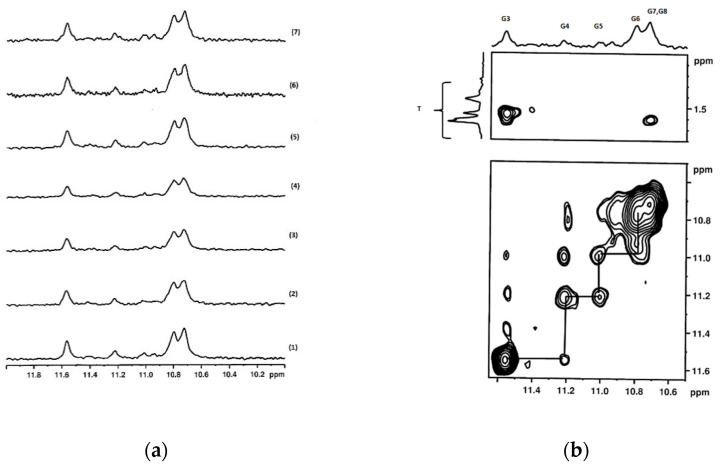
(**a**) Imino proton NMR spectra of oligonucleotide H2 (G-PO-HCV-L235). (1) Sample before heating. (2) Sample after heating at 90 °C for 1 min and move the sample to the magnet for NMR acquisition (approx. at 50 °C) (3) after 13 min, (4) after 30 min, (5) after 50 min, (6) after 65 min, and (7) after 5 months. (**b**) Imino and methyl protons region of 2D-NOESY spectrum of H2 (G-PO-HCV-L235) at 25 °C in H_2_O/D_2_O (9:1), PBS buffer (pH 7.4).

Oligonucleotide H2 (G-PO-HCV-L235) was dissolved in PBS buffer, pH 7.4 and the resulting solution was heated at 90 °C and cooled down to room temperature. Several NMR spectra were taken at different times. Figure 2a shows the NMR spectra of the imino protons region. In all of them, the presence of the imino protons characteristic of a G-quadruplex formation can be observed. The presence of these signals is observed just dissolving the sample. The formation of a G-tetrad gives rise to characteristic guanine imino protons (H1), which exhibit their chemical shifts within the range of 10–12 ppm, as compared to 13–14 ppm for those involved in Watson–Crick base pairing. Guanine imino protons in a G-quadruplex also exchange more slowly with solvent than the counterparts in a Watson–Crick duplex. The imino protons of guanines in the center G-tetrad exchange very slowly with the solvent and remain detected long after dissolving the sample in D_2_O solution [30]. Annealing (heating and cooling) the solution shows the presence of imino protons all the time, indicating that the formation of the G-quadruplex in the short 17 mers is very fast, as they do not change with the time (Figure 2). The guanine protons displayed the characteristic sequential imino H1/H1 and H1/H8 NOE interactions [31], thus confirming the formation quadruplex structure (Figure 2b).

A similar experiment was done with the longer oligonucleotide L3, but in this case the signals at the imino protons regions were too small to analyze (data not shown).

#### 2.2.3. Characterization of G-Quadruplex Formation by Gel Electrophoresis

**Figure 3:**
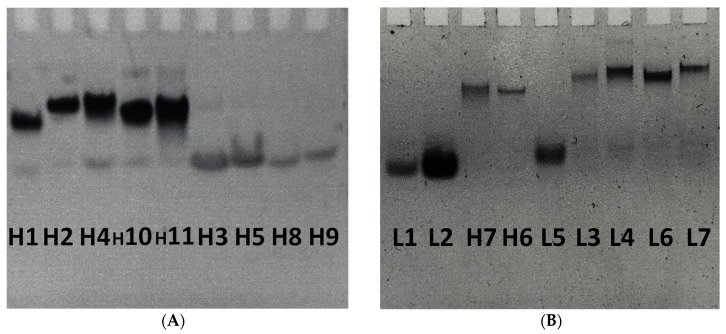
Native 12% PAGE of TBE 1X supplemented with 100 mM KCl using the same running buffer at 150 Watts maintaining a 20 °C for 4–5 h; stained with SYBR Green. (**A**) Short HIV G-rich sequences (H1, H2, H4, H10, H11) versus A-rich control sequences (H3, H5, H8, H9). (**B**) G-rich sequences carrying antiluciferase sequence (L3, L4, L6, L7) versus control single stranded sequences (L1, L2, L5) compared with short G-rich sequences (H6, H7).

Next, the G-quadruplex sequences were analyzed by native gel electrophoresis. Figure 3 shows the presence of single bands in the lanes with G-rich sequences that are retarded from the single-stranded control sequences in agreement with G-quadruplex formation (Figure 3A,B).

### 2.3. ASGPR Receptor Binding Studies

**Figure 4:**
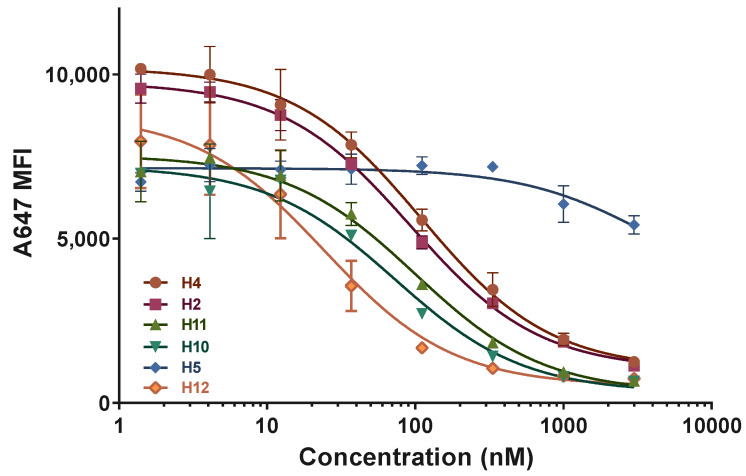
Affinity binding to ASGPR in primary mouse hepatocytes. Oligonucleotides derived from 17 mer antiviral sequence.

Next, the affinity binding of G-rich oligonucleotides to asialoglycoprotein receptors (ASGPR) were analyzed by a flow cytometry based competitive binding assay using mouse hepatocytes [12,14] and determining the inhibitory constant (KI). For comparison the oligonucleotide H12 (A-PO-HCV-L96) carrying the triantennary GalNAc was included in addition to G-rich oligonucleotides (G-PO-HCV-L193 (H4), G-PO-HCV-L235 (H2), G-PS-HCV-L193 (H10) and G-PS-HCV-L193 (H11) and a control A-rich sequence that is not able to form tetrameric G-quadruplex (A-PO-HCV-L193, H5). Figure 4 shows the displacement curves of all the GalNAc oligonucleotides from where a KI is estimated (Figure 4 and Table 3). The best affinity was found for the triantennary GalNAc (A-PO-L96, 10.4 nM) followed by the G-rich oligonucleotides carrying the shorter GalNAc linker (L235) (31.3 and 39.9 nM). Then, the G-rich oligonucleotides carrying the longer GalNAc linker (L193) followed with a KI of 42.8 and 47.2 nM. In both cases the oligonucleotide carrying phosphorothioate linkages had a slight better affinity. The affinity to ASGPR of the control single-stranded A-rich sequence was not possible to determine at concentrations tested. These results demonstrate the benefit of tetramerization by increasing ASGPR binding close to the affinity values observed for the triantennary GalNAc using G-rich oligonucleotides carrying a single monomeric GalNAc ligand.

**Figure 5:**
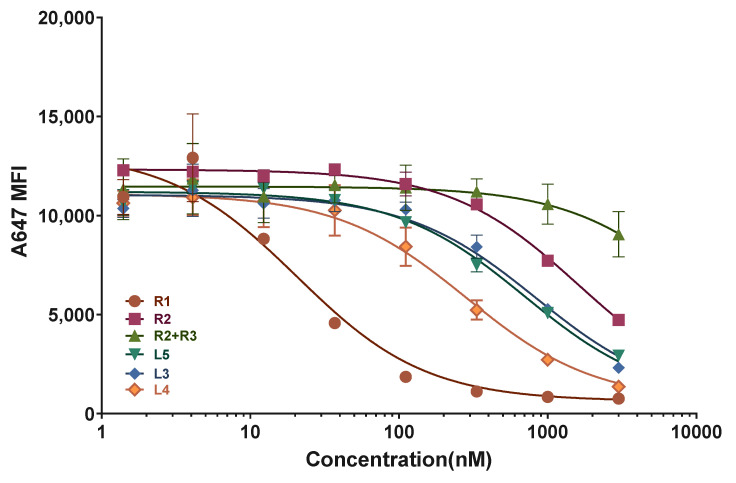
Affinity binding to ASGPR in primary mouse hepatocytes of gapmers and RNA derivatives carrying the anti-luciferase and anti-mTTR (Table S2) sequences.

The binding affinities to ASGPR of GalNAc-functionalized gapmers and RNA oligonucleotides linked to a TG_6_T sequence are shown in Figure 5 and Table 4. In this case we observed a loss of the affinity to ASGPR. Single-stranded and double stranded RNA carrying the TG_6_T sequence and one single monomeric GalNAc ligand have very low affinity to ASGPR (Sense-TG_6_T-L193 696 nM, Duplex-TG_6_T-L193 N/D). Gapmers (L3 and L4) have slightly better affinity especially L4 (G_6_-Luc-L193, 121 nM), but still far from the RNA carrying a triantennary GalNAc ligand R1 (sense-L96, 8.8 nM). There is a clear loss in binding affinity when the G-quadruplex sequence is next to the GalNAc ligand (L3, Luc-G6-L193, 357.8 nM) becoming of the same magnitude that the T-rich sequence that cannot form a quadruplex (L5, Luc-T_4_-L193, 300.7 nM). These results may indicate that when the oligonucleotide attached to the TG_6_T sequence becomes longer and complex the tetramerization becomes more difficult and the G-quadruplex structure dissociates rapidly to trimeric, dimeric and monomeric species that have less affinity for ASGPR. Although the gel retardation data show G-quadruplex formation, these quadruplexes may undergo to dissociation upon dilution.

### 2.4. Toxicity Assays

Prior the evaluation of internalization and antisense inhibition of luciferase, we evaluate the potential toxicity of GalNAc-oligonucleotides by using MTT assay at 60 nM, 120 nM and 300 nM oligonucleotide concentration after 24 h. Results are shown in Figure S5. Most of the compounds were not toxic, as all the MTT values were 80% or higher in HeLa cells (cervix cancer cells) and HepG2 (hepatic cells).

### 2.5. Analysis of the Stabilityof Oligonucleotides towards Snake Venom Phosphodiesterase and 10% FBS

Selected G-rich oligonucleotides (H6, H7, L4 and L7) were incubated with a solution of snake venom phosphodiesterase and a 10% FBS solution (experimental conditions used in cell studies). Results are shown in supplementary materials (Figure S4A,B). H6 (FAM-G-PO-HCV-L235) and H7 (FAM-G-PO-HCV-L193) G-rich oligonucleotides incubated with snake venom phosphodiesterase show some stability as the spots corresponding to the full length H6 and H7 are clearly visible, but disappeared between 8–24 h. In 10% FBS two groups of spots are seen: one having the mobility of the spots seen in the treatment snake venom phosphodiesterase and a retarded band that may correspond to the oligonucleotide complexed with serum proteins that are unchanged over the time of incubation (Figure S4A).

In the snake venom phosphodiesterase treatment of L4 (G_6_-Luc-GalNAc) and L7 (FAM-Luc-G6-GalNAc), the spots corresponding to the full length L4 and L7 are clearly visible after 24 h confirming the stability of these highly modified oligonucleotides (gapmers with full phosphorothioate linkages). In 10% FBS, only a main retarded band is seen that may correspond to the oligonucleotide complexed with serum proteins that are unchanged over the time of incubation (Figure S4B). These data indicate that these oligonucleotides are more stable than the H series because they carry phosphorothioate and 2′-O-methyl residues and these modifications increase binding of oligonucleotides to serum proteins that may protect oligonucleotides from degradation.

### 2.6. Internalization Assay

**Figure 6:**
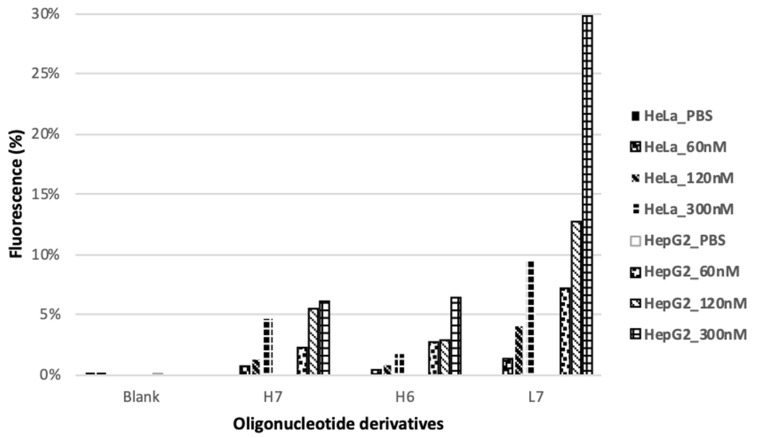
Cytometry assay evaluating the internalization of H7 (L193), H6 (L235), L7 (FAM-G_6_-Luc, FAM derivative of L3) at 60, 120 and 300 nM in HeLa and HepG2 cell lines.

The internalization of fluoresceine (FAM)-labelled G-rich GalNAc-oligonucleotides (H6, H7 and L7) were measured with a cell cytometer. Results are shown in Figure 6. Oligonucleotide L7 is the oligonucleotide whose internalization is higher in both HeLa and HepG2 cell lines. In fact, the internalization magnitude in HepG2 cells of the G-quadruplex compound is by a factor of 3 higher than in HeLa cells at same conditions so, this secondary structure push up the internalization process in HepG2 cells.

Independently analyzing the three compounds, on the one hand, oligonucleotide H7 in HeLa cells go from 1% green fluorescence intensity internalization to around 5% at 300 nM. On the other hand, in the HepG2 cell line, the green fluorescence intensity is slightly higher by going from around 2% to 6%. Oligonucleotide H6, a shorter linker than H7, it slims the internalization down in HeLa cells, but in HepG2 cells it can be seen a blip between low concentrations and the higher concentration of the compost. Finally, looking deeper on the results obtained with the FAM-G_6_-Luc (L7) compound, the differences between the two cell lines are very large. In HeLa cells, percentage of internalization goes from 3% at 60 nM to 10% at 300 nM. Then, in HepG2 cells goes from 7% to 30%.

### 2.7. Antisense Studies

**Figure 7:**
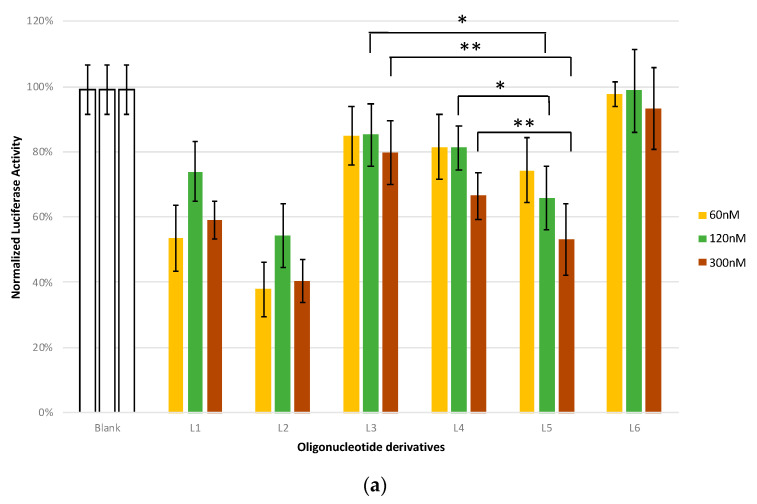

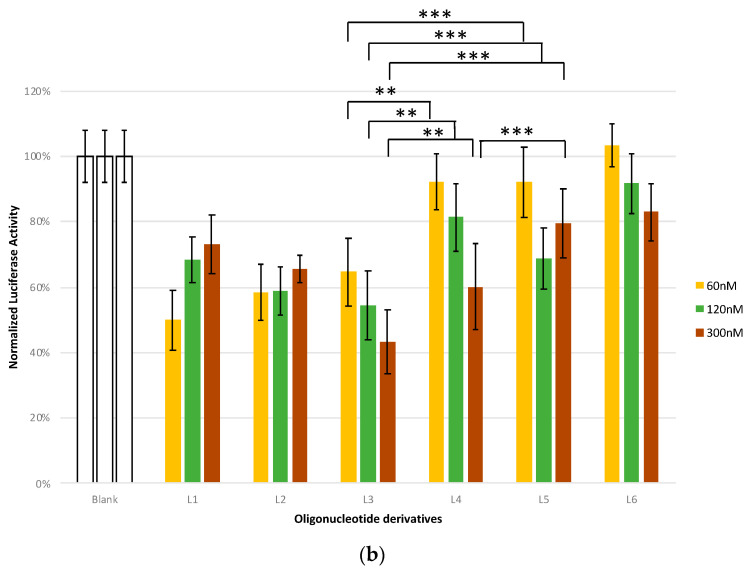
Luciferase inhibitory assay of [L1–L5] in HeLa (**a**) and HepG2 (**b**) cell lines, using L6 as a scrambled negative control and L1 (ASO) and L2 (Gapmer) transfected with lipofectamine as the positive control. Oligonucleotides carrying GalNAc are administrated without transfecting agent. * corresponds to *t*-test results with * *p* ≤ 0.1, ** *p* ≤ 0.05, *** *p* ≤ 0.01 significantly different within each indicated pair.

Next the luciferase silencing activity of antisense oligonucleotides was evaluated in HeLa and HepG2 cell lines. G-rich oligonucleotides carrying the antiluciferase gapmer sequence L3 (Luc-G_6_-L193) and L4 (G_6_-Luc-L193) were compared to T-rich oligonucleotide L5 (Luc-T_4_-L193) and the scrambled G-rich sequence L6 (Scr-Luc-G_6_-L193). As a positive control we used L1 (ASO) and L2 (Gapmer) transfected with Lipofectamine 2000. In the following graphs (Figure 7), the luciferase inhibition assay is represented in gymnotic conditions for the GalNAc-oligonucleotides. In HeLa cells (Figure 7a), the luciferase inhibitory effect of the G-rich oligonucleotides (L3 and L4) is low compared with the ASO and Gapmer controls transfected with Lipofectamine that inhibits around 70%. In Hela cells the product that has the best inhibitory properties is the L5 that is not able to form a G-quadruplex. This is in agreement with the lack of ASGPR as cellular uptake that is more favorable for the smallest oligonucleotide (L5) without the possibility of G-quadruplex tetramerization.

On the contrary the analysis of the luciferase assay results in HepG2 cell line (Figure 7b) shows the reverse situation. In this case the G-rich oligonucleotide L3 (Luc-G_6_-L193) is the most efficient antisense luciferase inhibitor, followed by L4 (G_6_-Luc-L193) that near 50% luciferase inhibition at 300 nM in gymnotic conditions reaching a very significant difference (*p* < 0.001) at 120 nM. In HepG2 cells, the smaller oligonucleotide has low inhibitory properties presenting an erratic shape from 60 nM to 300 nM concentration.

Comparing the inhibitory data in HepG2 cells with the results on ASGPR affinity data in mouse primary hepatocytes, a reverse effect is observed. Specifically, L3 (Luc-G_6_-L193) has higher inhibitory properties in HepG2 cells than L4 (G_6_-Luc-L193), but a lower ASGPR affinity in mouse primary hepatocytes. On the other, the luciferase inhibition data are in agreement with the cytometry assays that evaluate the internalization of L7 (FAM-Luc-G_6_-L193) that is the fluorescent version of L3. Figure 6 clearly demonstrated the high internalization efficacy of L7 in HepG2 cells in agreement of the high luciferase inhibitory properties of L3.

## 3. Discussion

The last five years have witnessed the approval of several oligonucleotides for human use [1,2]. A major success in this field is the development of the active targeting system based in the use of the triantennary GalNAc ligand directly conjugated to the nucleic acid part [32]. This ligand directs the therapeutic oligonucleotides to hepatocytes [12]. For these reasons, there is an interest for the synthesis of these valuable oligonucleotides. It has been proposed that a potential simplification on the synthesis of tri-GalNAc oligonucleotides is the stepwise addition of three monomeric GalNAc units at the 3′-end [13,14]. In this work, we have studied an alternative method aiming to simplify the synthetic process that consists in the use of the tetramerization of individual monomeric GalNAc-oligonucleotides by adding a short G-rich sequence (TGGGGGT) that form a parallel G-quadruplex. Monomeric GalNAc-oligonucleotides are easier to prepare than the triantennary GalNAc and the addition of eight extra deoxynucleotides will still be a clear simplification of the preparation of these valuable oligonucleotide conjugates. We hypothesized that the formation of the tetramer will have a similar effect to the addition of a triantennary GalNAc derivative. In addition, the presence of the G-quadruplex may also have a beneficial effect in cellular uptake as it has been hypothesized that G-quadruplex may bind to serum proteins and to certain protein membrane receptors overexpressed in cancer cells [18,19,20].

For these reasons we have previously studied the gene inhibitory properties of antisense oligonucleotides linked to lipid-modified G-quadruplex forming oligonucleotides [21,22]. Analysis of gene expression showed that the formation of self-assembled G-quadruplex nanostructures did not disrupt the antisense mechanism and flow cytometry analyses confirmed that antisense G-quadruplex nanostructures were efficiently taken up by cancer cells. In addition we found that some lipid–functionalized G-quadruplexes are effective antiviral compounds against Hepatitis C [17]. Moreover, floxuridine oligomers linked to parallel G-quadruplex were able to deliver fluoropyrimidines to cancer cells acting as prodrugs [23].

In this work, we studied the generation of parallel G-quadruplex functionalized with a monomeric GalNAc unit at the 3′- end and their potential in the selective targeting of antisense oligonucleotide against *Renilla* luciferase gene. To this end, two types of sequences were selected. First, we incorporated a GalNAc unit at the 3′- end of the 17 mer HIV-1 sequence [17] instead of the lipid moiety. The formation of a self-assembled parallel G-quadruplex was confirmed through various biophysical techniques such as circular dichroism, nuclear magnetic resonance, polyacrylamide electrophoresis and denaturation curves. In this work, we did measure the NMR spectra of the GalNAc-17 mer before and after annealing, observing that the NMR signals assigned to the imino protons of the G-quadruplex were present all the time, indicating the presence of quadruplex in all conditions. This is contrasted with the slow formation kinetics of parallel G-quadruplexes described by several authors [17,29]. The kinetics of tetramolecular quadruplex formation has mainly been studied in TG_4_T oligonucleotides [29]. These studies have also shown that association is slow and concentration-dependent as well as some other factors [29]. In addition, it has been described that the association rate can be accelerated by the presence of 8-modified guanine derivatives such as 8-bromo-G [33] or 8-amino-G [34]. There are no studies with TG_6_T in NMR experimental conditions because it is harder to quantify the kinetic parameters, as TG_6_T does not dissociate at 90 °C [29]. However, Mergny et al. described that the longer the G-tract, the faster the association and that the addition of one tetrad from TG_4_T introduces a 10-fold larger association rate [29]. One should expect that the association rates of TG_6_T will become 100-fold larger than TG_4_T. In addition, we have to consider that NMR experiments use relatively high concentrations compared with UV studies. The observed acceleration of quadruplex formation observed in our NMR experiments may also respond to the high concentration used in the experimental conditions, or because of the higher stability of the TG_6_T quadruplex. Finally, the effect of the presence of the extra nucleotides or the linkers with GalNAc has never been studied. Therefore, our study may indicate that there is an interesting story to be revealed. From the practical view in the biological experiments, we prepared a concentrated solution a couple of days before to make sure that all the quadruplex is formed without taking into account the faster association rates of the G-rich oligonucleotides presented in this work.

As expected for the formation of tetramers, the affinity binding of GalNAc-G-rich-17 mers to ASGPR (KI: 31.3–47.2 nM) were found to have a lower affinity than a single-stranded oligonucleotide functionalized with the triantennary GalNAc (A-PO-L96, KI 10.4 nM), but higher than the affinity to ASGPR of a control single-stranded A-rich sequence that was not possible to determine at concentrations tested. This result confirmed that tetramerization of monofunctionalized GalNAc G-rich sequences does indeed increase the affinity to ASGPR most probably by emulating the rapid internalization via the clathrin-mediated pathway described for the multifunctional triantennary oligonucleotides [12,15]. We have prepared two different GalNAc derivatives with different length (L193 and L235) as well as oligonucleotides carrying two phosphorothioate linkages at the terminal 3′ and 5′- ends, but the analysis of the ASGPR binding affinity resulted in values of the same range (KI: 31.3–47.2 nM). Although a slight higher affinity is observed for oligonucleotides carrying phosphorothioate linkages (KI: 31.3 and 42.8 vs. 39.9 and 47.2 nM) and carrying the shorter linker L235 (31.3 and 39.9 vs. 42.8 and 47.2).

Next we prepared a series of oligonucleotides that combined a gapmer sequence against *Renilla* luciferase carrying 2′-O-methyl-RNA modifications at the last four positions of the 3′- and 5′-ends and phosphorothioate linkages with a G-quadruplex forming TG_6_T sequence with phosphodiester linkages. In these cases, the characterization of the G-quadruplex is more challenging due to the complexity of the molecules, but the analysis of polyacrylamide electrophoresis clearly showed the presence of the G-quadruplex as a major species. Unfortunately, the affinity binding of GalNAc-G-rich oligonucleotides to ASGPR decrease more than one order of magnitude (KI: 8.8 vs. 121 and 357 nM). In addition, duplex formation abolishes binding of GalNAc-G-rich oligonucleotides to ASGPR, precluding the potential use of tetramerization for GalNAc active hepatocyte targeting for siRNA and other double-stranded oligonucleotides such as microRNA mimetics.

The biological properties of the hybrid gapmer/G-quadruplex molecules were evaluated in two cell lines (HepG2, HeLa) demonstrating the absence of toxicity. The analysis of the stability of hybrid gapmer /G-quadruplex molecules towards degradation in 10% PBS indicates that these oligonucleotides are more stable than the H series because they carry phosphorothioate and 2′-O-methyl residues and these modifications increase binding of oligonucleotides to serum proteins that may protect oligonucleotides from degradation.

The internalization preferences of GalNAc-quadruplexes to hepatic cells were also demonstrated and specifically the cellular uptake of oligonucleotide carrying the antisense sequence (FAM-Luc-G_6_-GalNAc, L7) and the G-rich sequence goes from 9% in HeLa cells to 30% in HepG2 cells (at 300 nM concentration). Consequently, to the good internalization properties of the gapmer-G-quadruplex hybrid molecules L3 and L4, we observed an enhancement of the luciferase inhibition in HepG2 cell lines versus HeLa cells, L3 being the more active gapmer in HepG2 cells.

All together, we have demonstrated the possibility of obtaining multifunctional nanoassemblies by simple hybridization of monofunctionalized oligonucleotides. This approach has been extensively studied in the bibliography and holds promise for future developments [35]. For example, three oligonucleotides designed to form a triplex were functionalized with a short coiled peptide that interacts between them, stabilizing the triplex structure [36]. Similarly, G-quadruplex formation has been shown to direct the assembly of two peptide strands generating two-loop structures on top of the G-quadruplex. This approach can be used with homo and hetero peptide sequences [37]. G-rich oligonucleotides designed to form parallel G-quadruplex functionalized with hydrophobic group are able to tetramerize resulting in multifunctionalized G-quadruplex with affinity to viral proteins [17,38] and/or cell membranes [21,22,23]. The strategy here described can be extended for the hepatic delivery of antiproliferative and antiviral nucleosides by the prodrug strategy described recently [23].

## 4. Materials and Methods

Lipofectamine 2000 was purchased from Invitrogen. Dulbecco’s Modified Eagle’s Medium (DMEM) was supplemented with 10% heat-inactive fetal serum bovine (FBS). DMEM, PBS buffer and distilled water (DNAse/RNAse free) were purchased from Gibco (Waltham, MA, USA). Luciferase assay kits were acquired from Promega (Madison, WI, USA). Luminescence was measured in a Promega Glomax Multidetection System instrument. Flow cytometer analyses were carried out in a Guava^®^ easyCyte. Luciferase plasmids (p-GL3 and p-RL) were extracted of growing up E. coli transfected previously with the corresponding plasmid and purified with Qiagen Giga plasmid purification kit purchased from Qiagen.

### 4.1. Synthesis of GalNac Oligonucleotide Conjugates and Controls

Control oligonucleotides were synthesized on an Applied Biosystems DNA synthesizer using solid-phase phosphoramidite chemistry. These oligonucleotides were 5′-TG_6_T-3′, 5′-T_8_-3′ the anti-luciferase phosphorothioate oligonucleotide 5′-CsGsTsTsTsCsCsTsTsTsGsTsTsCsTsGsGsA-3′, and the gapmer sequence 5′-CsGsUsUsTsCsCsTsTsTsGsTsTsCsUsGsGsA-3′ where the underlined nucleotides are 2′-O-methyl-RNA and contain phosphorothioate linkages. The antisense phosphorothioate and the gapmer oligonucleotides are complementary to the mRNA of the *Renilla* luciferase gene which target to a predominant accessible site between 20 and 40 nt of the luciferase gene [27].

Oligonucleotides carrying *N*-acetylgalactosamine (GalNAc) shown in Table 1 and Table 2 have been synthesized using solid-phase phosphoramidite chemistry. For the introduction of the GalNAc at the 3′-end two special solid supports were prepared functionalized with *O*-tetraacetyl-*N*-acetyl galactosamine connected to 4-hydroxyprolinol through alkyl chains of different lengths (L235 and L193) (Scheme 1). The synthesis of solid supports functionalized with these GalNAc derivatives has been reported in detail previously [13]. All oligonucleotides were purified by HPLC, and the major peak was characterized by mass spectrometry (Table S1).

### 4.2. G-Quadruplex Formation

All G-rich and control oligonucleotides were treated in the same conditions to reduce the differences between samples. First, oligonucleotides were quantified, aliquoted appropriately for the different experiments and concentrated to dryness. Oligonucleotides used in CD, gel electrophoresis and in cell experiments were dissolved in 100 mM PBS (pH 7.4). In all cases, solutions were annealed by heating at 93 °C for 2 min in a thermo-block and slow cooling down to room temperature (2–3 days). The resulting oligonucleotide solutions were stored at 4 °C until used.

### 4.3. Circular Dichroism

CD spectra of the annealed solutions were registered between either from 205 to 320 nm or from 220 to 320 nm at 20 °C in PBS 1× buffer. CD thermal denaturation experiment of the G-quadruplex-forming oligonucleotide (G-PO-HCV-L235, H2) (4–5 μM) was performed in a range of temperature between 20 and 85 °C using a heating rate of 0.8 °C min^−1^ and monitoring the ellipticity at 263 nm.

### 4.4. Nuclear Magnetic Resonance (NMR) Experiments

The NMR sample of H2 (G-PO-HCV-L235, 2 mg in 0.55 mL, 0.63 mM) was prepared in of PBS buffer (0.01 M phosphate buffer, 0.0027 M KCl and 0.137 M NaCl) (H_2_O:D_2_O 9:1), pH 7.4. The oligonucleotide was heated to 90 °C for 1 min and then cooled at room temperature overnight. ^1^H-NMR spectra were acquired at 25 °C at different times after the heating with a Bruker AV600, 600 MHz spectrometer, equipped with a TXI probe with z-gradient, and processed with TOPSPIN 2.1 software. Two-dimensional NOESY spectra were acquired with mixing time of 300 ms and 2D TOCSY spectra with mixing time of 60 ms.

### 4.5. Polyacrylamide Electrophoresis Assays

A native polyacrylamide gel electrophoresis (PAGE) was carried out using 12% (*v*/*v*) acrylamide to characterize the mobility of the assembled oligonucleotides. Samples were dissolved in PBS and the gel was run in 1× TBE buffer supplemented with 100 mM KCl at 150 V (12% PAGE) for approximately 4–5 h maintaining a fixed temperature of 20 °C. SYBR green (20 µL in 200 mL 1× TBE) was used to stain the DNA bands and then images were taken using Fujifilm LAS-1000 Intelligent Dark Box II as well as IR LAS-1000 Lite v1.2. The ladder used was a solution containing Bromophenol Blue and Xylene Cyanol for visual tracking of oligonucleotides migration during the electrophoretic process.

### 4.6. Affinity Binding to ASGPR Receptors in Primary Mouse Hepatocytes

ASGPR competitive binding was evaluated as previously described [14]. Briefly, 20 nM triantennary GalNAc-conjugated, Alexa647-labeled siRNA, described previously [12], and 3 µM to 1.4 nM of relevant oligonucleotide were premixed and then co-incubated with 1 × 10^5^ of viable plateable primary mouse (CD-1) cryopreserved hepatocytes (ThermoFisher Scientific, Waltham, MA, USA) in Dulbecco’s Modified Eagle Medium (DMEM) with 2% bovine serum albumin (BSA). Samples were incubated at 4 °C for 15 min, then washed twice with 2% BSA in Dulbecco’s Phosphate-Buffered Saline with magnesium and calcium (DPBS). Cells were suspended in a solution of 2% BSA in DPBS with 2 µg/mL propidium iodide and analyzed on a BD LSRII flow cytometer. Hepatocytes were gated by size using forward scatter and side scatter, and dead cells stained with propidium iodide were excluded from analysis. Median fluorescence intensity of the Triantennary GalNAc-conjugated, Alexa647-labeled siRNA was quantified (A647 MFI). Data were analyzed using FlowJo and KI was calculated in GraphPad Prism using a derived Michaelis-Menten equation for competitive inhibition.

### 4.7. MTT Assays

HeLa and HepG2 cells were regularly passaged to maintain exponential growth and proprieties. Cells were seeded for 8000 cells into each well of a 96-well cell culture plate. Then, cells were incubated overnight at 37 °C and 5% CO_2_ in DMEM supplemented with 10% FBS. After that, the oligonucleotide conjugates were added and the cells incubated for 24 h at increasing concentrations (60, 120 and 300 nM) in 200 mL of new DMEM (10% FBS). PBS buffer was used as a control. Before that time, growth medium was removed and cells were washed with PBS (200 mL), so all compounds not internalized were removed and 200 mL of fresh DMEM (10% FBS) was added to incubated the cells again for 12 h more at 37 °C. Next day, using a MTT dye solution (25 mL; 5 mg mL^−1^) was added per well and cells were incubated for two additional hours. Medium was removed and DMSO (100 mL) was added to dissolve formazan crystals formed and the absorbance of the solution was measured in a plate reader. Data was analyzed in a excel sheet. The MTT assay was repeated 12 times in six independent experiments in Hela cells and 10 times in 5 independent experiments in HepG2 cells.

### 4.8. Flow Cytometry

HeLa and HepG2 cells (80,000 and 100,000 cells per well, respectively) were seeded on 24-well cell culture plate. Then, they were incubated overnight at 37 °C and 5% CO_2_ in DMEM supplemented with 10% FBS. Next day, fluorescein-labelled oligonucleotides were added and the cells were incubated for 24 h. After that time, cells were washed with PBS (500 μL) and harvested with trypsin (200 mL) at 37 °C for some minutes. DMEM (800 μL) was added and cells were centrifuged (3.0 rcf 8 min). DMEM supernatants were removed and pellets were washed with 800 μL of PBS to analyze them in a cytometer instrument. For each sample, 5000 events were collected in a selected gate (R1) that corresponds to the cell population (each line was analyzed with a different template). Guava soft software Incite surface was used to analyze the relationship between fluorescently labeled and unlabeled cell populations and quantify the percentages of each type of cells.

### 4.9. Luciferase Assays

HeLa and HepG2 cells were seeded in the same 24-well plate as the previous experiment, but in a confluence of 100,000 and 200,000 cells per well, respectively. After overnight incubation at 37 °C and 5% CO_2_ in DMEM + 10% FBS, cells were transfected with a solution of two luciferase plasmids *Renilla* and Firefly luciferase (pRL (10 ng/µL) and pGL3 (100 ng/µL)) in Optimem buffer (without plasma proteins) using lipofectamine 2000 to internalize the plasmids into the different cell lines. After 4 h of incubation, cells were thoroughly washed, and the incubation with the Luc-oligonucleotides at 60–120–300 nM was done without lipofectamine, so differences between cells will affect to the internalization of the drug. All these experiments were done in a final volume of 600 μL. An ASO as control at the same concentration was used for comparison purposes. Transfections were performed in triplicate and for 24 h. After this time, cells were washed again to eliminate all the Luc-compound not internalized and frozen. Finally, lysates were analyzed by comparing the luminescence of both Firefly and *Renilla* luciferase protein according to manufacturer’s protocol. The luciferase assay was repeated 30 times in 12 independent experiments in Hela cells and 30 times in 8 independent experiments in HepG2 cells.

### 4.10. Analysis of the Stability of Oligonucleotides towards Snake Venom Phosphodiesterase and 10% FBS

Briefly, 10 μL of the oligonucleotide at 5 μM were dissolved in 100 µL of a mixture of Tris·HCl buffer (100 mM), MgCl_2_ solution (100 mM) and 1 μL of phosphodiesterase I from *Crotalus adamanteus* venom (USB) was added and incubated in 37 °C. Then, 5 µL of the solution was removed (every certain time) and added to 15 μL of urea solution (8M) and heated to 85 °C for 5 min and stored at the freeze. Finally, the different samples were analyzed by denaturing (8M urea) 12% polyacrylamide gel electrophoresis. Similarly, 10 μL of the oligonucleotide at 5 μM were dissolved in 10% FBS and incubated at 37 °C. As before, 5 µL of the solution was removed and added to 15 μL of urea solution (8M) and heated to 85 °C for 5 min and stored at the freeze. Then, the different samples were analyzed in denaturing (8M urea) 12% polyacrylamide gel electrophoresis.

## 5. Conclusions

There is a large interest in the development of defined DNA nanostructures carrying multifunctional ligands that are recognized by membrane receptors to achieve more efficient delivery systems for the fast-growing field of therapeutic oligonucleotides. To this end, we addressed the potential use of the tetramerization of G-rich sequences to build novel methods to achieve the multivalency of the well-known GalNAc ligands using simple oligonucleotides carrying a single GalNAc ligand. To this end, several G-rich oligonucleotides carrying the TGGGGGGT sequence were prepared carrying one GalNAc molecule per oligonucleotide. The synthesis of these monofunctionalized GalNAc-oligonucleotides is easier than the standard triantennary GalNAc derivatives due to the complexity of the synthesis of the triantennary GalNAc ligand. We studied three potential tetrameric G-quadruplexes carrying four GalNAc molecules by tetramerization of monofunctionalized GalNAc G-rich oligonucleotide: I. Tetravalent GalNAc linked to the HVC sequence (H series, Table 1). II. Tetravalent GalNAc linked to an antisense oligonucleotide against luciferase gene (Gapmer, L series, Table 2). III. Tetravalent GalNAc linked to a siRNA (R series, Table S2). The study show that structures I and II can be observed and may be responsible for an increased affinity to ASGPR and an increased antisense activity in hepatocytes while structure III most probably is not formed (Figure S1). The formation of a self-assembled parallel G-quadruplex in structures I and II was confirmed through various biophysical techniques. Binding experiments to asialoglycoprotein receptors (ASGPR) show that structure I achieve the best affinity binding to ASGPR and clearly better than single-stranded control oligonucleotides that cannot form G-quadruplex demonstrating that powerful advantages of multifunctionality. Binding experiments to ASGPR in structures II show a less efficient ASGPR binding, but still better than single-stranded control oligonucleotides. In addition some differences are observed depending of the relative position of the therapeutic cargo. The internalization preferences of GalNAc-quadruplexes to hepatic cells were also demonstrated by cell cytometry. As well as the enhancement of the luciferase inhibition under gymnotic conditions using the luciferase assay in the HepG2 cell line. All together demonstrates that tetramerization of G-rich oligonucleotides can be used to obtain the beneficial effects of multivalent GalNAc functionalization although in long oligonucleotides as well as duplex siRNA tetramerization is less efficient probably by competing duplex formation.

## Figures and Tables

**Table 1:** Oligonucleotide derivatives from 17 mer antiviral sequence.

N	Name	Sequence (5′-3′)	Backbone	GalNAc
H1	control	TTGGGGGGTACAGTGCA	PO	-
H2	G-PO-HCV-L235	TTGGGGGGTACAGTGCA-L235	PO	L235
H3	A-PO-HCV-L235	TTGAAAGGTACAGTGCA-L235	PO	L235
H4	G-PO-HCV-L193	TTGGGGGGTACAGTGCA-L193	PO	L193
H5	A-PO-HCV-L193	TTGAAAGGTACAGTGCA-L193	PO	L193
H6	FAM-G-PO-HCV-L235	FAM-TTGGGGGGTACAGTGCA-L235	PO	L235
H7	FAM-G-PO-HCV-L193	FAM-TTGGGGGGTACAGTGCA-L193	PO	L193
H8	A-PS-HCV-L235	TsTsGAAAGGTACAGsTsGsCsA-L235	PO/PS	L235
H9	A-PS-HCV-L193	TsTsGAAAGGTACAGsTsGsCsA-L193	PO/PS	L193
H10	G-PS-HCV-L235	TsTsGGGGGGTACAGsTsGsCsA-L235	PO/PS	L235
H11	G-PS-HCV-L193	TsTsGGGGGGTACAGsTsGsCsA-L193	PO/PS	L193
H12	A-PO-HCV-L96	TTGAAAGGTACAGTGCA-L96	PO	L96

s indicates the position of the phosphorothioate linkages.

**Table 2:** Oligonucleotide derivatives carrying anti-luciferase sequence.

N	Name	Sequence (5′-3′)	Backbone	GalNAc
L1	ASO control	CsGsTsTsTsCsCsTsTsTsGsTsTsCsTsGsGsA	PS	-
L2	Gapmer control	CsGsUsUsTsCsCsTsTsTsGsTsTsCsUsGsGsA	PS	-
L3	Luc-G_6_-GalNAc	CsGsUsUsTsCsCsTsTsTsGsTsTsCsUsGsGsATGGGGGGT-L193	PO,PS	L193
L4	G_6_-Luc-GalNAc	TGGGGGGTCsGsUsUsTsCsCsTsTsTsGsTsTsCsUsGsGsAs-L193	PO,PS	L193
L5	Luc-T_4_-GalNAc	CsGsUsUsTsCsCsTsTsTsGsTsTsCsUsGsGsATTTT-L193	PO,PS	L193
L6	Scr-Luc-G_6_-GalNAc	CsUsGsUsCsTsGsAsCsGsTsTsCsTsUsUsGsUsTGGGGGGT-L193	PO,PS	L193
L7	FAM-Luc-G_6_-GalNAc	FAM-sCsUsGsUsTsCsCsTsTsTsGsTsTsCsUsGsGsAsTTGGGGGGT-L193	PO,PS	L193
L8	T_8_	TTTTTTTT	PO	-

s indicates the position of the phosphorothioate linkages. Underline nucleotides are 2-O-methyl-RNA derivatives.

**Table 3:** Binding affinity to ASGPR using a mouse primary hepatocytes fluorescence-based assay [12,14].

N	Name	KI (nM)	Stdev
H4	G-PO-HCV-L193	47.2	7.3
H2	G-PO-HCV-L235	39.9	3.2
H11	G-PS-HCV-L193	42.8	7.6
H10	G-PS-HCV-L235	31.3	7.2
H12	A-PO-HCV-L96	10.4	3.2
H5	A-PO-HCV-L193	N/D ^1^	N/D ^1^

^1^ N/D = not determined at concentrations tested.

**Table 4:** Binding affinity to ASGPR using a mouse primary hepatocytes fluorescence-based assay [12,14].

N	Motif	KI (nM)	Stdev
R1	Sense-L96	8.8	2.2
R2	Sense-TG_6_T-L193	696.0	37.2
R2 + R3	Duplex-TG_6_T-L193	N/D ^1^	N/D ^1^
L5	Luc-T_4_-L193	300.7	23.9
L3	Luc-G_6_-L193	357.8	48.5
L4	G_6_-Luc-L193	121.1	17.6

^1^ N/D = not determined at concentrations tested.

## Data Availability

The data presented in this study are available on request from the corresponding author.

## Supplementary Materials

The following supporting information can be downloaded at: https://www.mdpi.com/1420-3049/27/12/3944/s1, Figure S1. Schematic representation of the potential tetrameric G-quadruplexes. Table S1: Sequences and mass spectra of oligonucleotide derivatives prepared in this work. Table S2: Oligonucleotide RNA derivatives carrying anti-mTTR siRNA sequence. Figure S2: Melting curves on Gquadruplex sequences. Figure S3: CD spectra of G-rich and control oligonucleotides. Figure S4: Analysis of stability of G-rich oligonucleotides to FBS and phosphodiesterase. Figure S5: MTT assay in HeLa (a), and HepG2 (b) cells.

## Sample Availability

Samples of the oligonucleotides prepared in this work are available from the authors upon request to the corresponding author.
