# Correction: Clua et al. Properties of Parallel Tetramolecular G-Quadruplex Carrying N-Acetylgalactosamine as Potential Enhancer for Oligonucleotide Delivery to Hepatocytes. *Molecules* 2022, *27*, 3944

**DOI:** 10.3390/molecules28010098

**Published:** 2022-12-23

**Authors:** Anna Clua, Santiago Grijalvo, Namrata Erande, Swati Gupta, Kristina Yucius, Raimundo Gargallo, Stefania Mazzini, Muthiah Manoharan, Ramon Eritja

**Affiliations:** 1 Institute for Advanced Chemistry of Catalonia (IQAC-CSIC), Jordi Girona 18-26, E-08034 Barcelona, Spain; 2 Networking Center on Bioengineering, Biomaterials and Nanomedicine (CIBER-BBN), E-08034 Barcelona, Spain; 3 Alnylam Pharmaceuticals, 300 Third Street, Cambridge, MA 02142, USA; 4 Department of Chemical Engineering and Analytical Chemistry, University of Barcelona, Martí i Franquès 1-11, E-08028 Barcelona, Spain; 5 DEFENS-Dipartimento Di Scienze per Gli Alimenti, la Nutrizione e l’Ambiente, Università degli Studi di Milano, Via Celoria, 2, 20133 Milan, Italy

In the original article [1], there was a mistake in the position of one of the OH of N-acetylgalactosamine. In the original article [1], the OH was in an equatorial position and it should be in an axial position. This mistake affected Scheme 1 and Scheme 2. Below, we include the figures from the original manuscript and the figures that are corrected. We apologize for this unintentional mistake. 

Original Scheme 1:

**Scheme 1:**
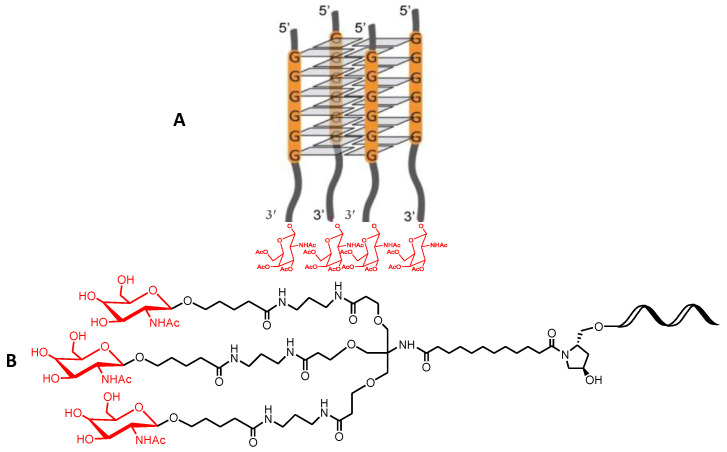
Chemical schemes of potential multifunctional GalNAc derivatives including (**A**) tetrameric G-quadruplexes studied in this work and (**B**) the triantennary GalNAc.

This should be replaced with the following:

**Scheme 1:**
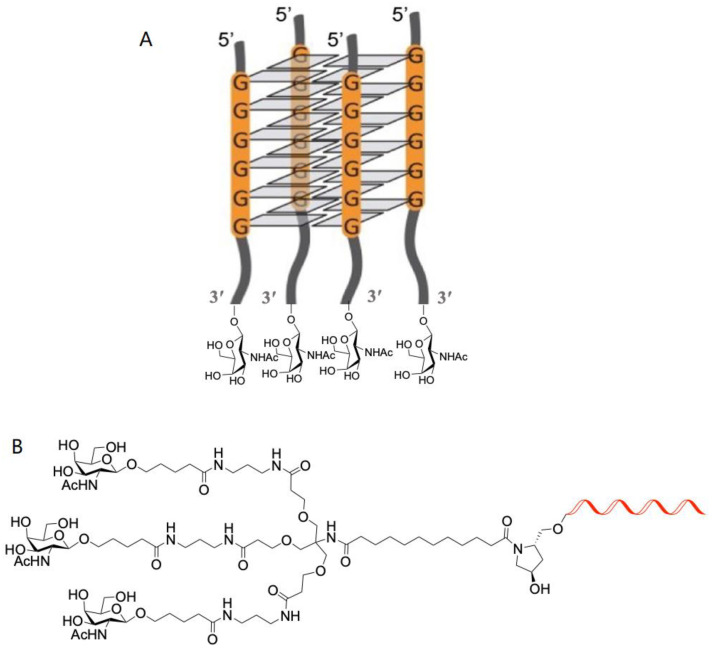
Chemical schemes of potential multifunctional GalNAc derivatives including (**A**) tetrameric G-quadruplexes studied in this work and (**B**) the triantennary GalNAc.

Original Scheme 2:

**Scheme 2:**
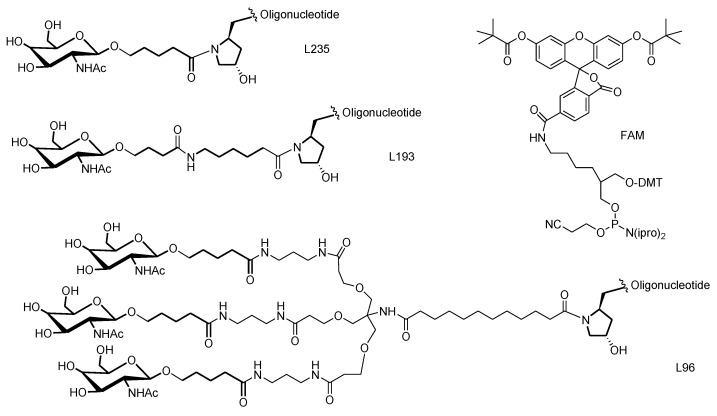
Chemical structures of the GalNAc and FAM ligands used in this work.

This should be replaced with the following:

**Scheme 2:**
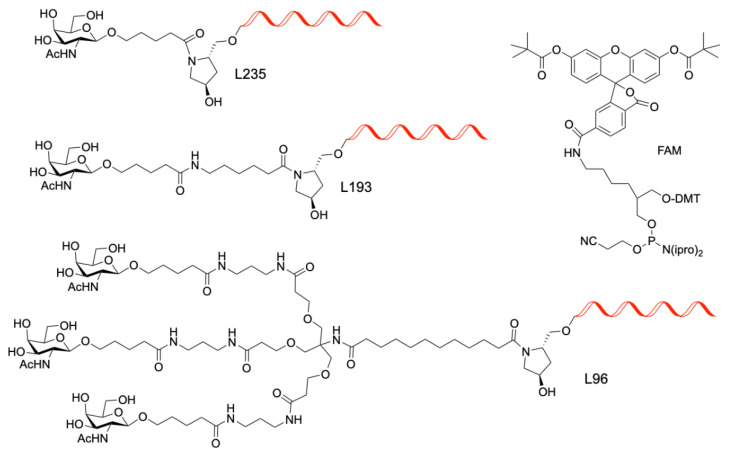
Chemical structures of the GalNAc and FAM ligands used in this work.
