# Supplementary material for: Properties of Parallel Tetramolecular G-Quadruplex Carrying N-Acetylgalactosamine as Potential Enhancer for Oligonucleotide Delivery to Hepatocytes
Source: Molecules. 2022 Jun 20;27(12):3944. doi: 10.3390/molecules27123944 (PMC9228010; doi:10.3390/molecules27123944)

Supporting Information

Properties of parallel tetramolecular G-quadruplex carrying N-acetylgalactosamine as potential enhancers for oligonucleotide delivery to hepatocytes.

Anna Clua[1,2], Santiago Grijalvo[1,2], Namrata Erande[3], Swati Gupta[3], Kristina Yucius[3], Raimundo Gargallo[4], Stefania Mazzini[5], Muthiah Manoharan[3], and Ramon Eritja[1,2,*]

[1]Institute for Advanced Chemistry of Catalonia (IQAC-CSIC), Jordi Girona 18-26, E-08034 Barcelona, Spain; acvtnt@cid.csic.es (A.C.); sgrgma@cid.csic.es (S.G.) [2]Networking Center on Bioengineering, Biomaterials and Nanomedicine (CIBER-BBN), E-08034 Barcelona [3]Alnylam Pharmaceuticals, 300 Third Street, Cambridge, Massachusetts 02142, United States; nd.erande@gmail.com (N.E.); sgupta@alnylam.com (S.G); kyucius@alnylam.com (K.Y.); mmanoharan@alnylam.com (M.M.) [4]University of Barcelona, Department of Chemical Engineering and Analytical Chemistry, Martí i Franquès 1-11, E-08028 Barcelona, Spain; raimon_gargallo@ub.edu (R.G) [5]Università degli Studi di Milano, DEFENS-Dipartimento Di Scienze per Gli Alimenti, la Nutrizione e l'Ambiente, Via Celoria, 2 Italia-20133, Milano, Italy; stefania.mazzini@unimi.it (S.M.)

Corresponding Author

* Email; recgma@cid.csic.es (R. E).

Table of contents:

1. **Figure S1**. Schematic representation of the potential tetrameric G-quadruplexes. Page 3
2. **Table S1**. Sequences and mass spectra of oligonucleotide derivatives prepared in this work.. Page 4
3. **Table S2**. Oligonucleotide RNA derivatives carrying anti-mTTR siRNA sequence. Page 4
4. **Figure S2**. Melting curves on G-quadruplex sequence (G-PO-HCV-L235). Page 5
5. **Figure S3**. CD spectra of G-rich and control oligonucleotides Page 6
6. **Figure S4**. Analysis of stability of G-rich oligonucleotides to FBS and phosphodiesterase. Page 7
7. **Figure S5**. MTT assay in HeLa (a), and HepG2 (b) cells. Page 9

Figure S1. Schematic representation of the potential tetrameric G-quadruplexes carrying four GalNAc molecules by tetramerization of monofunctionalized GalNAc G-rich oligonucleotide. **I.** Tetravalent GalNAc linked to HVC sequence (H series, Table 1). **II.** Tetravalent GalNAc linked to antisense oligonucleotide (Gapmer, L series, Table 2). **III**. Tetravalent GalNAc linked to an siRNA (R series, Table S2). The study shows that structures I and II can be observed and may be responsible of an increased affinity to ASGPR and an increased antisense activity in hepatocytes while structure III most probably is not formed.

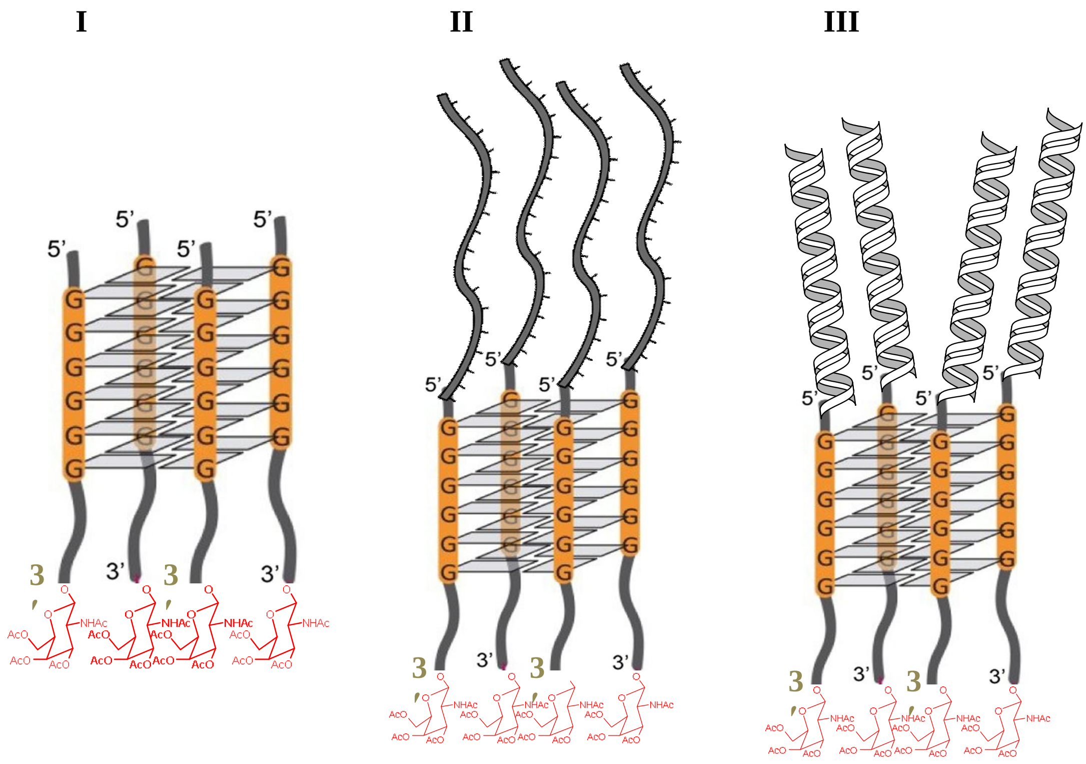

Table S1. Sequences and Mass spectra of Oligonucleotide derivatives prepared in this work.

N	Name	Sequence (5'-3')	MW Exp.	MW Found
H1	control	TTGGGGGGTACAGTGCA	5304	5295
H2	G-PO-HCV-L235	TTGGGGGGTACAGTGCA-L235	5786	5776
H3	A-PO-HCV-L235	TTGAAAGGTACAGTGCA-L235	5738	5727
H4	G-PO-HCV-L193	TTGGGGGGTACAGTGCA-L193	5885	5889
H5	A-PO-HCV-L193	TTGAAAGGTACAGTGCA-L193	5837	5841
H6	FAM-G-PO-HCV-L235	FAM-TTGGGGGGTACAGTGCA-L235	6353	6342
H7	FAM-G-PO-HCV-L193	FAM-TTGGGGGGTACAGTGCA-L193	6452	6454
H8	A-PS-HCV-L235	TsTsGAAAGGTACAGsTsGsCsA-L235	5834	5824
H9	A-PS-HCV-L193	TsTsGAAAGGTACAGsTsGsCsA-L193	5933	5936
H10	G-PS-HCV-L235	TsTsGGGGGGTACAGsTsGsCsA-L235	5885	5872
H11	G-PS-HCV-L193	TsTsGGGGGGTACAGsTsGsCsA-L193	5981	5986
H12	A-PO-HCV-L96	TTGAAAGGTACAGTGCA-L96	7043	nd
L1	ASO control	CsGsTsTsTsCsCsTsTsTsGsTsTsCsTsGsGsA	5732	5720
L2	Gapmer control	CsGsUsUsTsCsCsTsTsTsGsTsTsCsUsGsGsA	5912	5920
L3	Luc_G_6_GalNAc	CsGsUsUsTsCsCsTsTsTsGsTsTsCsUsGsGsAT GGGGGGT-L193	9075	Broad 9200 (M+nNa)
L4	G_6_Luc_GalNAc	TGGGGGGTCsGsUsUsTsCsCsTsTsTsGsTsTs CsUsGsGsA-L193	9075	9115 (M+2Na)
L5	Luc_T_GalNAc	CGUUTCCTTTGTTCUGGATTTT-L193	7773	7728 / 7770
L6	Scr_Luc_G_6_GalNAc	CsUsGsUsCsTsGsAsCsGsTsTsCsTsUsUsGsUs TGGGGGGT-L193	9055	Broad 9500 (M+nNa)
L7	FAM_Luc_G_6_GalNAc	FAM-CsUsGsUsTsCsCsTsTsTsGsTsTsCsUsGsGsAT TGGGGGGT-L193	9642	Broad 9800 (M+nNa)
L8	T_8	TTTTTTTT	2370	2365

nd: not determined. "s" indicate the position of the phosphorothioate linkages. Underlined nucleotides indicate 2'-O-methyl-RNA derivatives

Table S2. Oligonucleotide RNA derivatives carrying anti-mTTR siRNA sequence. Small letters (a, u, g, c) indicate 2'-O-Me nucleotide; capital letters with F (Af, Uf, Gf, Cf) indicate 2'-fluororibonucleotide; capital letters (A, G, U, C) indicate RNA nucleotides: capital letters with d in (dA, dT, dG, dC) indicate 2'-deoxy nucleotides; "s" in (as, us,gs, cs, Afs, Ufs, Gfs, Cfs, dAs) indicate the position of the phosphorothioate linkages.

N	Name	Sequence (5'-3')	GalNAc
R1	A117799 sense	Afs-as-Cf-a-Gf-u-Gf-u-Uf-Cf-Uf-u-Gf-c-Uf-c-Uf-a-Uf-a-Af-L96	L96
R2	A1411192 sense	Afs-as-Cf-a-Gf-u-Gf-u-Uf-Cf-Uf-u-Gf-c-Uf-c-Uf-a-Uf-a-Af-dTdTdTdTdGdGdGdGdGdGdT-L193	L193
R3	A117800 antisense	Us-Ufs-a-Uf-a-Gf-a-Gf-c-Af-a-g-a-Af-c-Af-c-Uf-g-Uf-us-us-u	---

Figure S2. Melting curves on G-rich sequence H2 (G-PO-HCV-L235). CD spectra shows minor changes with increasing temperature. The CD signal at 265 nm does not change with increasing temperature up to 90 ºC. The same results were found for the G-rich sequences H4 (G-PO-HCV-L193), H10 (G-PS-HCV-L235) and H11 (G-PS-HCV-L193). Working concentration (5 μM) in 10 mM lithium cacodylate buffer pH 7.2 supplemented with 0.11M NaCl. Samples were heated up to 90 ºC. All studied G-rich oligonucleotides were stable up to 90 ºC.

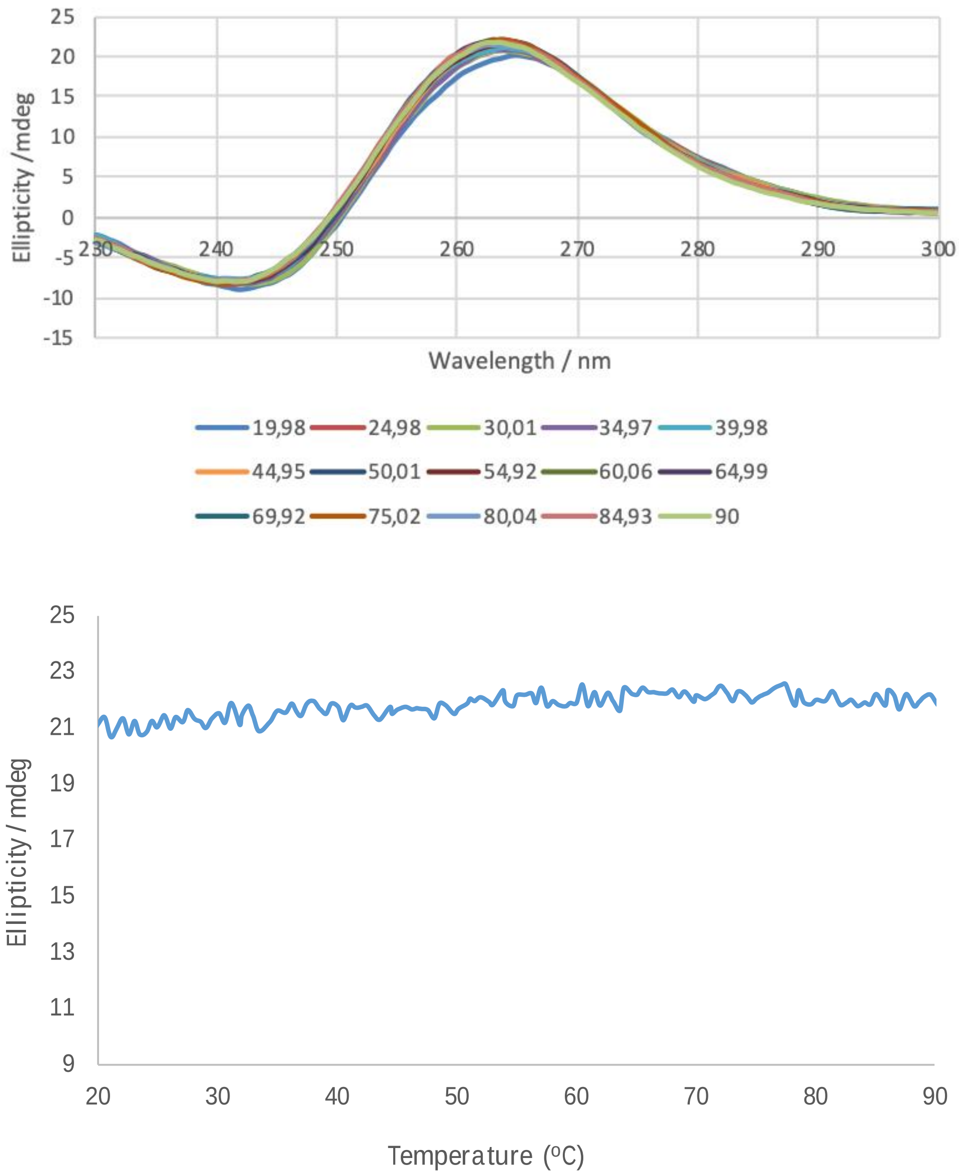

Figure S3. CD spectra of **A)** Oligonucleotides H2 (G-PO-HCV-L235), H3 (A-PO-HCV-L235), H4 (G-PO-HCV-L193), H10 (G-PS-HCV-L235), and H11 (G-PS-HCV-L193) and **B)** Oligonucleotides L3 (Luc_G_6_GalNAc), L4 (G_6_Luc_GalNAc), L5 (Luc_T_4_GalNAc), L6 (Scr_Luc_G_6_GalNAc). G-rich oligonucleotides show the maximum at 265 nm compared with non A-rich (H3) or T-rich (L5) control sequences that have the maximum at 275 nm. Working concentration (5 μM) in PBS 1x.

A)

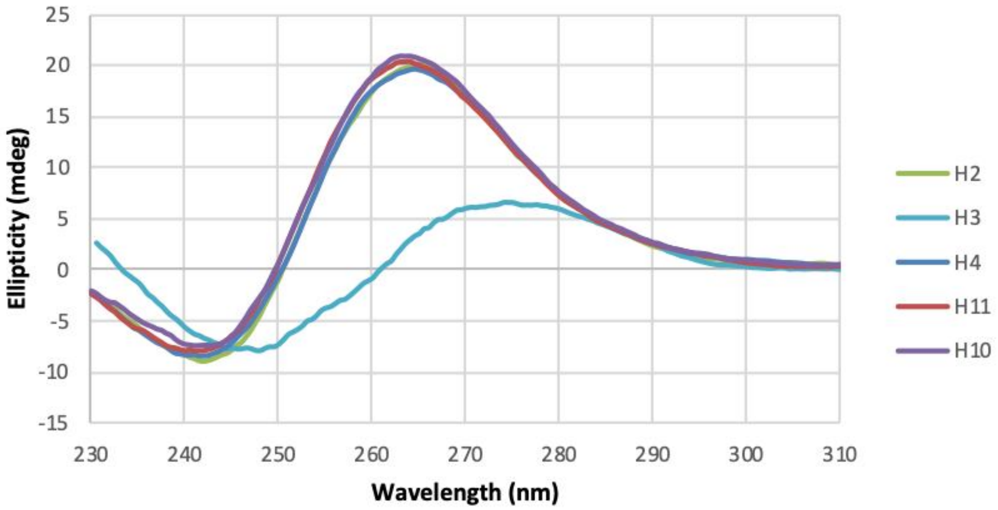

B)

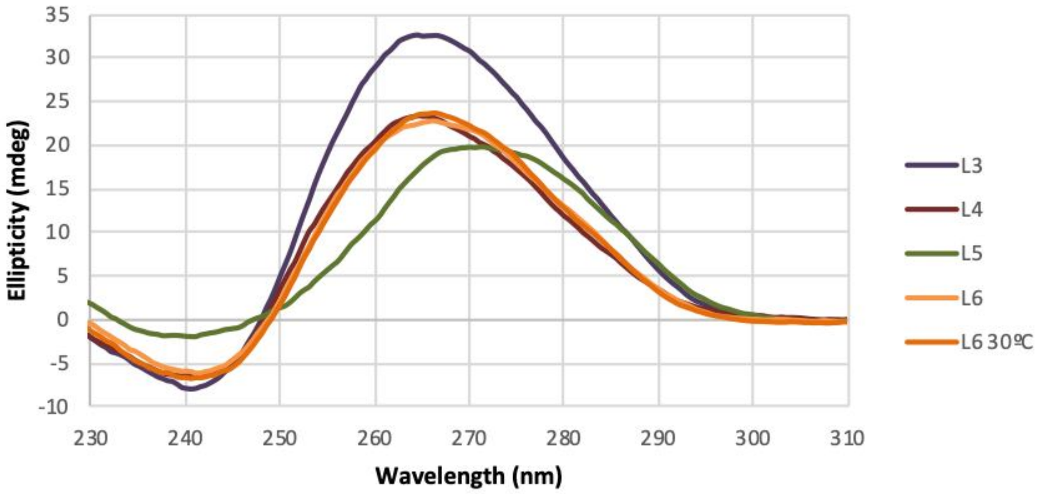

Figure S4. **A**. Analysis of stability of H6 (FAM-G-PO-HCV-L235) and H7 (FAM-G-PO-HCV-L193) G-rich oligonucleotides towards snake venom phosphodiesterase and 10% FBS (experimental conditions in cell studies). In snake venom phosphodiesterase, at 4 hr the spots corresponding to the full length H6 and H7 are clearly visible and disappeared between 8-24 hours. In 10% FBS two spots are seen: one having the mobility of the spots seen in snake venom phosphodiesterase that present a similar stability and a retarded band that may correspond to the oligonucleotide complexed with serum proteins that are unchanged over the time of incubation.

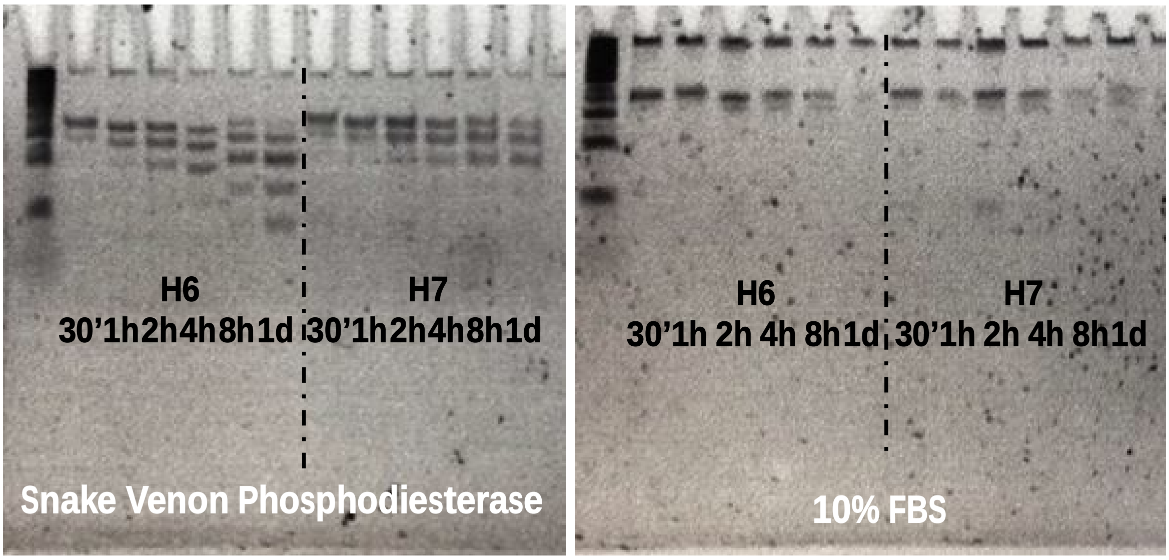

Figure S4. **B**. Analysis of stability of L4 (G_6_Luc_GalNAc) and L7 (FAM-Luc_G_6_GalNAc) G-rich oligonucleotides towards snake venom phosphodiesterase and 10% FBS (experimental conditions used in cell studies). In snake venom phosphodiesterase, the spots corresponding to the full length L4 and L7 are clearly visible after 24 hours confirming the stability of these highly modified oligonucleotides (gapmers with full phosphorothioate linkages). In 10% FBS only a main retarded band is seen that may correspond to the oligonucleotide complexed with serum proteins that are unchanged over the time of incubation. These data indicate that most probably these oligonucleotides carrying phosphorothioate and 2'-O-methyl residues bind to serum proteins that may protect the oligonucleotides from degradation.

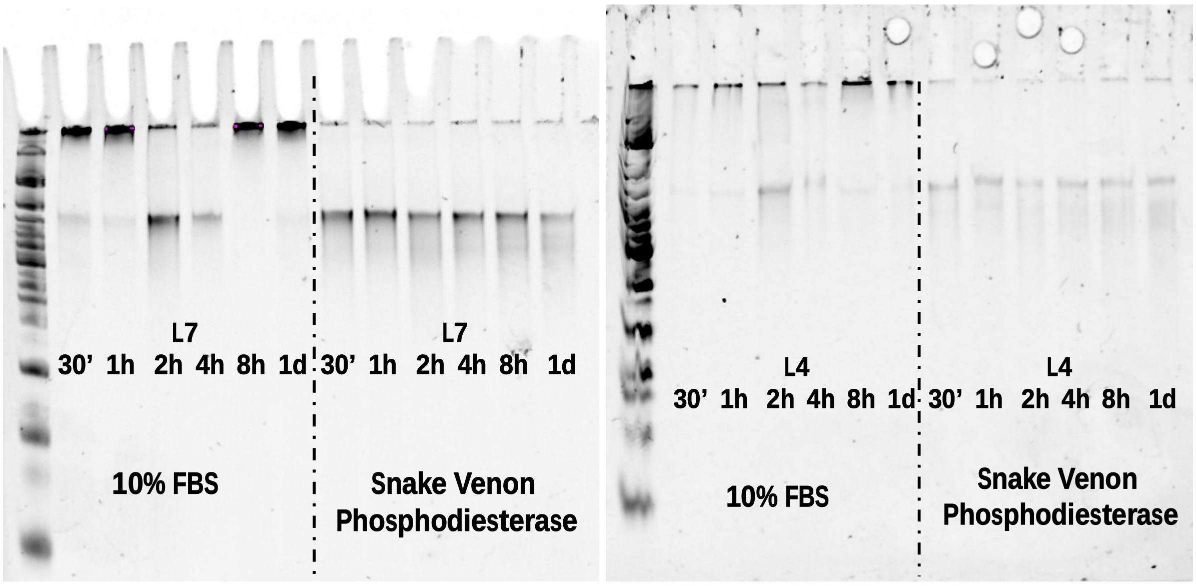

Figure S5. MTT assay in HeLa (a), and HepG2 (b) at 60, 120 and 300nM concentration of oligonucleotides. Luc_T_4_GalNAc (L5), Luc_G_6_GalNAc (L3), 5'_G_6_Luc_GalNAc (L4), FAM_Luc_G_6_ GalNAc (L7).

a)

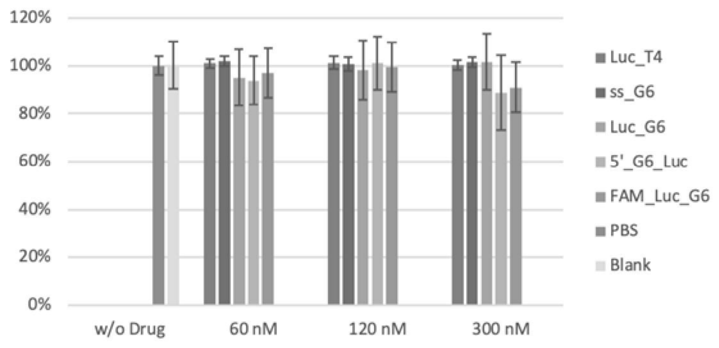

b)

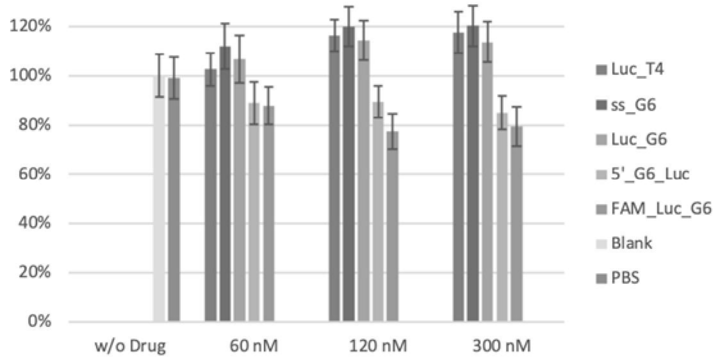

Supplement: Supplementary file 1 [file molecules-27-03944-s001.zip › molecules-1747415-supplementary.pdf]
